# EZH2‐mediated downregulation of miR‐155‐5p contributes to prostate cancer cell malignancy through SMAD2 and TAB2

**DOI:** 10.1002/kjm2.12936

**Published:** 2025-01-09

**Authors:** Zhi‐Jie Bai, Jia‐Yi Liu, Wen‐Zhou Xing, Hai‐Feng Wang

**Affiliations:** ^1^ Department of Urology Tianjin First Central Hospital Tianjin China

**Keywords:** EZH2, miR‐155‐5p, prostate cancer, SMAD2, TAB2

## Abstract

miR‐155 exhibits variable expression in different tumors and fulfills diverse biological roles. However, specific molecular mechanisms by which miR‐155‐5p, which is under‐expressed in prostate cancer (PCa), operates are yet to be elucidated. The role of the enhancer of zeste 2 (EZH2)/miR‐155‐5p axis in PCa was determined by using bioinformatics tools and performing luciferase reporter assay, chromatin immunoprecipitation PCR, CCK‐8 assays, cell migration and invasion assays, RNA isolation, reverse transcription quantity (RT‐qPCR) and Western blot. miR‐155‐5p expression would be reduced and promoter methylation would increase in PCa. After 5‐Aza‐CdR treatment and the integration of the upstream promoter of miR‐155‐5p into a pGL3‐basic/luciferase construct, fluorescence reporter analysis showed that promoter hypermethylation mediated the suppression of miR‐155‐5p in PCa. Furthermore, EZH2 attached to the miR‐155‐5p promoter and modulated its expression. EZH2 facilitated the suppression of miR‐155‐5p through enhanced H3K27me3 methylation, considerably affecting its expression. Through dual‐luciferase assays, SMAD2 and TAB2 were confirmed as downstream targets of miR‐155‐5p, regulating the PCa cellular phenotype governed by miR‐155‐5p. Lastly, 5‐Aza‐CdR regulated miR‐155‐5p expression by modulating its promoter methylation and influenced the malignant behavior of PCa cells. EZH2 promotes H3K27me3 methylation, repressing miR‐155‐5p expression, which subsequently upregulates the downstream targets SMAD2 and TAB2 and promotes PCa cell proliferation, epithelial–mesenchymal transition (EMT), migration and invasion.

## 1 INTRODUCTION

Prostate cancer (PCa) is a prevalent malignancy, especially in older males.^1^, ^2^ When the cancer spreads beyond the prostate to areas such as bones, it causes obvious clinical signs and complications, including bone pain, fractures, high calcium levels in the blood and compression of the spinal cord.^3^, ^4^, ^5^ Thus, clinical management for PCa is challenging and affects the quality of life and future health outcomes of patients with PCa. Hence, gaining a comprehensive knowledge of underlying molecular mechanisms in PCa and identifying key biomarkers for assessing its status and prognosis are critical to the formulation of strategies for early detecting and preventing PCa and personalized treatment approaches.

miR‐155 levels vary among different human solid tumors, influencing unique biological functions. In hepatocellular carcinoma, miR‐155‐5p level increases, facilitating epithelial–mesenchymal transition (EMT) and stem cell differentiation by suppressing TP53INP1 expression.^6^ Likewise, in breast malignancy, its elevated levels trigger EMT by reducing TGFBR2 expression and TGF‐β signaling pathway activation.^7^ However, a reduction in miR‐155‐5p level has been observed in the serum and tissue samples of individuals with PCa.^8^, ^9^ To date, specific molecular mechanisms underlying its downregulation in PCa are still not completely known.

DNA methylation is a complex phenomenon of epigenetic remodeling involving multiple interactions between chromatin and proteins, including polycomb repressive complexes 2 and 1 (PRC2 and PRC1).^10^, ^11^ Enhancer of zeste 2 (EZH2) is a component of the PRC2, which specifically participates in the covalent modification of histone tails, particularly H3K27me3.^12^ EZH2 facilitates CpG island (CGI) DNA methylation by recruiting DNA methyltransferases, thereby epigenetically regulating genes that determine cell fate.^13^ For instance, high EZH2 expression is closely linked to the malignant progression of PCa.^14^ EZH2 specifically regulates H3K27me3 to silence miR‐31 expression.^15^ Through bioinformatics analysis, potential CGIs were identified in the promoter region of miR‐155‐5p. Therefore, its low expression in PCa may be linked to DNA methylation in its promoter region.

In this investigation, we attempted to explore the role of the epigenetic silencing of miR‐155‐5p in PCa cell lines. EZH2 inhibited the expression of miR‐155‐5p in PCa by inducing the hypermethylation of its promoter region. This process resulted in the loss of the inhibitory effect of miR‐155‐5p on SMAD2 and TAB2, thereby promoting the malignant behavior of PCa cells.

## 2 MATERIALS AND METHODS

### 2.1 Cell culture

RWPE1, C4‐2, PC‐3, 22RV1 and DU145 were obtained from the American Type Culture Collection (USA). The cells were cultured in a DMEM enriched with 10% fetal bovine serum (Cat No. # 03.C16001DC, EallBio, Beijing, China) and 100 U/mL penicillin plus 100 mg/mL streptomycin (Cat No. # IA0340, Beijing Solarbio Science & Technology Co., Ltd., Beijing, China).

### 2.2 Cell transfection

Cell transfection was conducted utilizing Lipofectamine 3000 (Cat No. #L3000001, Invitrogen, Waltham, MA, USA) according to the manufacturer's instructions.

### 2.3 Prediction of miR‐155‐5p promoter and CpG islands

The enhanced prediction of miR‐155‐5p transcript upstream promoter regions was carried out using Promoter 2.0 (Server: https://services.healthtech.dtu.dk/services/Promoter-2.0/) and Neural Network Promoter Prediction (https://www.fruitfly.org/seq_tools/promoter.html). MethPrimer (http://www.urogene.org/methprimer/) was used in predicting the involvement of miR‐155‐5p in transcription through the presence of a CGI in the upstream promoter region.

### 2.4 Vector construction

The 1500‐bp upstream sequence of the miR‐155‐5p transcript was synthesized and constructed into a pGL3‐basic/luciferase vector by General Biology (Anhui) Co., Ltd. Subsequently, the pGL3‐miR‐155‐5p‐promoter‐luciferase (pGL3‐miR‐155‐5‐promoter‐luc) construct was obtained. The overexpression vectors for EZH2, SMAD2 and TAB2 were synthesized and constructed into the pcDNA3 vector by General Biology (Anhui) Co., Ltd. The knockdown plasmid for EZH2 was constructed by the same company, and relevant sequences were synthesized and constructed into the pSilencer 2.1‐U6‐Neo vector. Additionally, reporter vectors for SMAD2 and TAB2 3′UTRs were established, and wild‐type and mutant sequences were synthesized and constructed into pMIR‐REPORT™ luciferase. Furthermore, General Biology (Anhui) Co., Ltd. was commissioned to synthesize miR‐NC, miR‐155‐5p mimics, inhibitor‐NC or inhibitor‐miR‐155‐5p. The specific sequences can be found in Table S1.

### 2.5 RNA isolation and reverse transcription quantity (RT‐qPCR)

A Trizol kit was used in isolating total RNA (Cat No. # R1100, Beijing Solarbio Science & Technology Co., Ltd., Beijing, China), which was then diluted to 40 μg/mL and reverse‐transcribed into complementary DNA. A 25 μL PCR reaction system was prepared. The PCR conditions consisted of denaturation initially at 95°C for 5 min, followed by 40 amplification cycles at 95°C for 30 s, annealing at 60°C for 30 s, extension at 72°C for 30 s and a final extension step at 54°C for 10 min. Subsequently, the melting curve was generated, and the Ct value was determined along with the calculation of the relative expression level (2^−△△Ct^). Glyceraldehyde‐3‐phosphate dehydrogenase was employed as the internal reference for SAMD2 and TAB2, whereas U6 was utilized as the internal reference for miR‐155‐5p. The primers utilized are given in Table S2.

### 2.6 Chromatin immunoprecipitation assay

Chromatin immunoprecipitation (ChIP) assays were conducted utilizing a ChIP kit (Cat No. # abs50034, Absin Bioscience Inc., Shanghai, China) according to the provided protocol. Cells underwent fixation and cross‐linking with 1% formaldehyde. The nucleoprotein complexes were then fragmented to a length of 200–500 bases with ultrasound. Subsequently, immunoprecipitation was performed with an anti‐EZH2 antibody (Cat No. # 21800‐1‐AP, Proteintech, Wuhan, Hubei, China) at 4°C overnight. PCR was utilized for detecting the enrichment of DNA fragments containing miRNA‐binding sites in the upstream coding region of miR‐155‐5p. The primers used for detecting the upstream sequence of miR‐155‐5p are listed in Table S2.

### 2.7 CCK‐8 assays

PCa cells from each group that had undergone transfection or treatment were seeded onto 96‐well plates. Afterwards, the culture medium was substituted with a fresh medium consisting of CCK‐8 reagent at 0, 24, 48 and 72‐h time intervals. After a 1.5‐h incubation period, the absorbance was assessed in each well at 450‐nm wavelength with a microplate reader for the calculation of cell viability.

### 2.8 Cell migration and invasion assays

For migration detection, PC⁃3 and DU145 cells were harvested from each group and resuspended at 1 × 10^6^ cells/mL concentration. Afterwards, 200 μL of the suspension was inoculated into the upper chamber of Transwell. Cell medium with 20% serum was then introduced into the lower chamber, and culture was continued for 48 h. The cells in the lower chamber underwent fixation with formaldehyde and were subsequently subjected to staining with crystal violet dye. A light microscope was utilized for examining and counting the stained cells. For cell invasion detection, the upper chamber of Matrigel‐coated Transwell was utilized. The other steps were the same as those used in the migration experiment.

### 2.9 Luciferase reporter assay

Dual‐luciferase reporter assay was executed as previously detailed.^16^

### 2.10 Western blot analysis

Cells in each group were harvested and lysed using RIPA lysate (Cat No. # R0010, Beijing Solarbio Science & Technology Co., Ltd., Beijing, China) containing protease inhibitors for 20 min. The isolation of total protein was conducted through centrifugation at 12,000 rpm and 4°C for 15 min and quantified through BCA (Cat No. # PC0020, Beijing Solarbio Science & Technology Co., Ltd., Beijing, China). A protein loading buffer was added at a ratio of 4:1. The protein analysis was carried out through polyacrylamide gel electrophoresis, and the proteins were transferred onto a polyvinylidene fluoride membrane (Cat No. # 0000187216, 0.45 μm, Millipore Corp., Burlington, MA, USA). The membranes were blocked with 5% skim milk powder. Primary antibodies were separately added, and the samples were refrigerated at 4°C overnight. The primary antibodies encompassed EZH2 polyclonal antibody (1:5000, Cat No. # A2363, Abclonal, Wuhan, Hubei, China), H3K27me3 (1:1000, Cat No. # 21800‐1‐AP, Proteintech, Wuhan, Hubei, China), histone H3 (1:5000, Cat No. # A2348, Abclonal, Wuhan, Hubei, China), β‐tubulin (1:5000, Cat No. # 2146S, Cell Signaling Technology Inc., Boston, USA), E‐cadherin polyclonal antibody (1:1000, Cat No. # ab314063, Abcam, USA), vimentin (1:2000, Cat No. # PAB040Hu01, CLOUD‐CLONE CORP., Wuhan, Hubei, China), SMAD2 (1:1000, Cat No. # 5339, Cell Signaling Technology Inc., Boston, USA) and TAB2 (1:1000, Cat No. # 3745, Cell Signaling Technology Inc., Boston, USA). HRP‐labeled secondary antibody (1:5000, Cat No. #AS014, Abclonal, Wuhan, Hubei, China) was added the next day and subsequently incubated for 1 h at room temperature. Afterwards, an electrochemiluminescence solution was applied to develop the membrane. The relative expression of the protein was determined by utilizing a gel imaging analysis system.

### 2.11 Statistical analyses

The experiments were executed in triplicate and analyzed utilizing SPSS 21. One‐way analysis of variance was utilized for the comparative assessment of multiple samples, whereas an independent sample t‐test was utilized for the comparative assessment of the two samples. A *p* value of <0.05 was deemed as representing statistical significance.

## 3 RESULTS

### 3.1 Association of promoter methylation of miR‐155‐5p with its low expression

**FIGURE 1:**
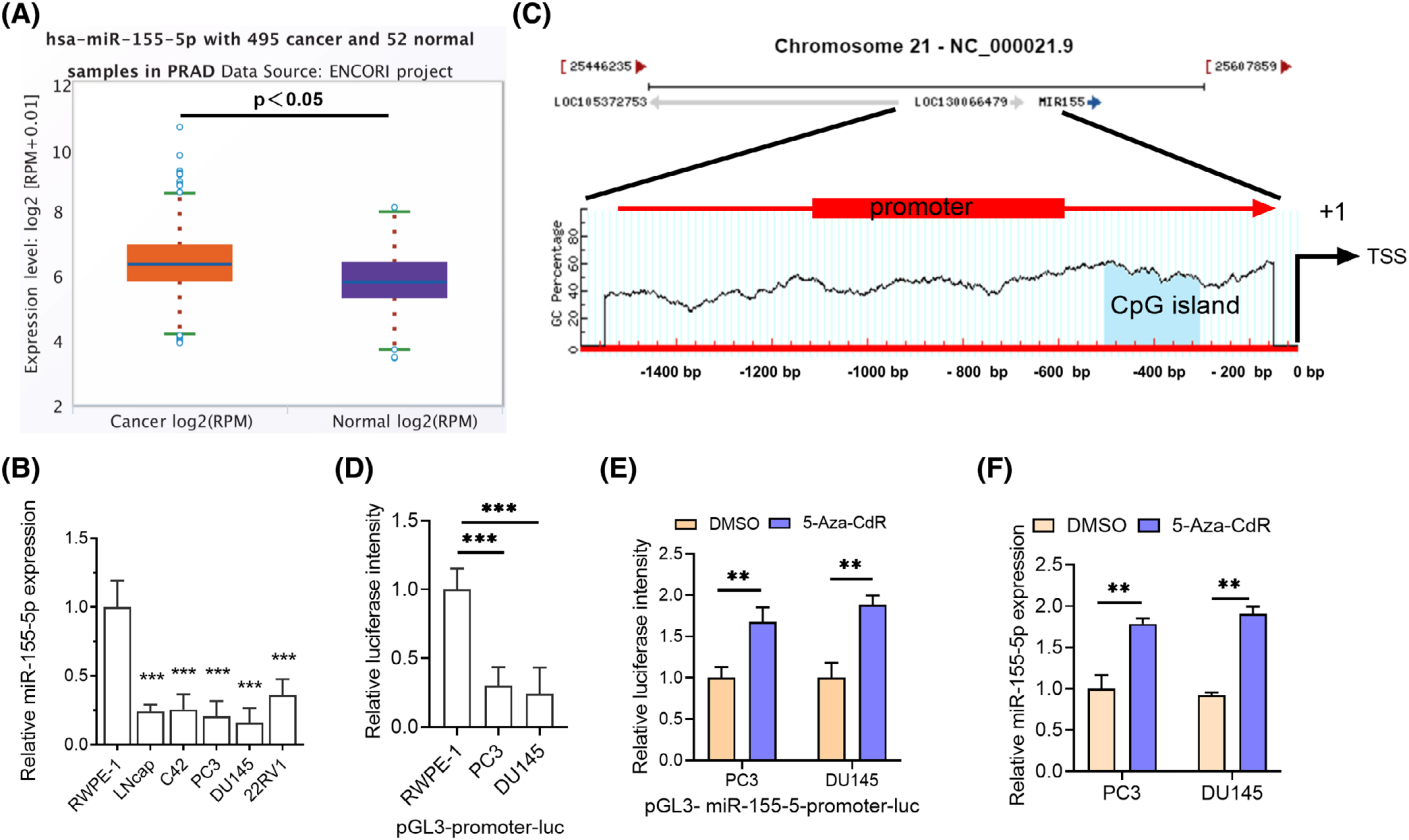
miR‐155‐5p exhibits decreased expression in PCa and is associated with promoter methylation. (A) Bioinformatics assessment of miR‐155‐5p level in PCa. (B) RT‐qPCR detection of miR‐155‐5p expression level in normal prostate cells and five PCa cell lines. (C) The bioinformatics website MethPrimer (http://www.urogene.org/methprimer/) was utilized for assessing miR‐155‐5p promoter methylation. (D) Luciferase reporter assay was utilized to detect miR‐155‐5p promoter activity in normal cells and PCa cell lines. (E) Luciferase reporter assay was utilized for detecting miR‐155‐5p promoter activity in PCa cells after 5‐Aza‐CdR (5 μM) treatment. (F) RT‐qPCR detection of miR‐155‐5p expression in PCa cells following treatment with 5‐Aza‐CdR. *p < 0.05, **p < 0.01, ***p < 0.001.

First, prediction analysis using the bioinformatics online tool starbase v 3.0 database revealed a considerable reduction in miR‐155‐5p level in PCa (Figure 1A). Subsequently, prostate normal epithelial cells (RWPE1) and 4 PCa cells (C4‐2, PC‐3, 22RV1, DU145) were examined, revealing a considerable reduction in miR‐155‐5p levels in the PCa cell lines relative to the normal epithelial cells (Figure 1B). Methylation analysis through the bioinformatics website MethPrimer indicated potential methylation in the upstream promoter CGI of miR‐155‐5p transcription (Figure 1C). Further investigation involved constructing a pGL3‐miR‐155‐5p‐promoter‐luciferase vector and transfecting RWPE1, PC‐3 and DU145 cells. The acquired data revealed reduced luciferase activity in the PCa cell lines, suggesting decreased promoter activity (Figure 1D). The transfection of PC‐3 and DU145 cells with pGL3‐miR‐155‐5 p‐promoter‐luc and subsequent treatment with 5‐Aza‐CdR increased luciferase activity, indicating methylation at the upstream promoter region where miR‐155‐5p was located (Figure 1E). Finally, treatment with 5‐Aza‐CdR increased endogenous miR‐1555p levels (Figure 1F). These findings implied that the hypermethylation of miR‐155‐5p promoter DNA mediated its downregulation in PCa.

### 3.2 EZH2 regulated miR‐155‐5p expression through H3K27me3 methylation

**FIGURE 2:**
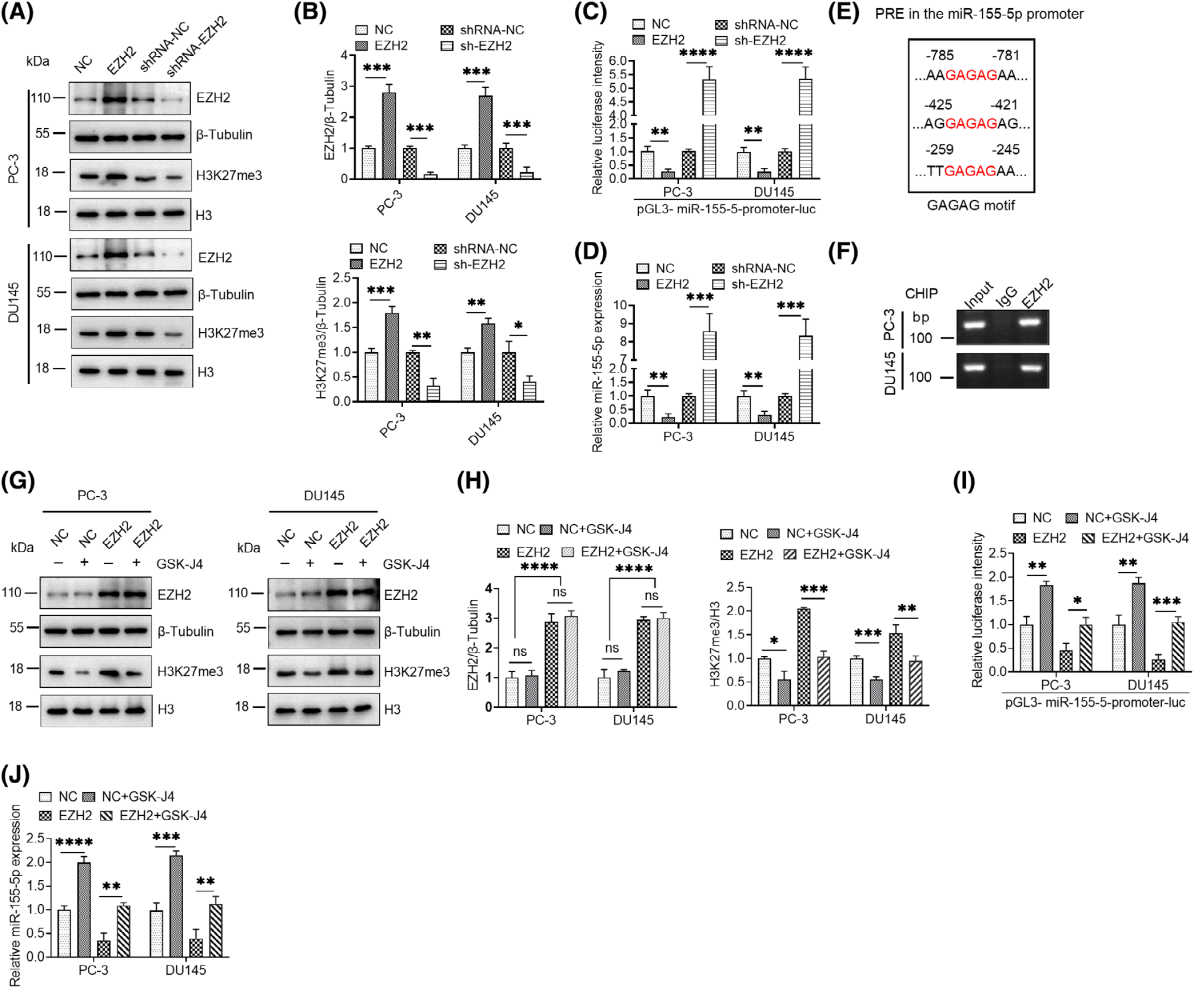
EZH2‐mediated H3K27me3 methylation regulates miR‐155‐5p expression. (A) After EZH2 was overexpressed or knocked down in PCa cells (DU145 and PC‐3), the relevant proteins were assessed via Western blot, determining their expression levels. (B) Quantitative analysis of the proteins given in (A). (C) miR‐155‐5p promoter activity was assessed using the luciferase reporter system in normal cells and PCa cell lines after EZH2 overexpression or knockdown in PC‐3 and DU145 PCa cells. (D) RT‐qPCR was utilized for detecting the level of miR‐155‐5p in PCa cells after EZH2 was overexpressed or knocked down in PC‐3 and DU145 cells. (E) Schematic of the core DNA motif (red) of the polycomb response element (PRE). (F) Chromatin immunoprecipitation PCR assay (ChIP‐PCR) was used in analyzing whether EZH2 is bound to the miR‐155‐5p promoter region. (G) PCa cells overexpressing EZH2 were treated with H3K27me3 inhibitor GSK‐J4 (1 μM), and the levels of expression of EZH2 and H3K27me3 proteins were detected by Western blot. (H) Quantification of the associated proteins given in (G). (I) PCa cells overexpressing EZH2 and miR‐155‐5p promoter reporter vector pGL3‐promoter‐luc after H3K27me3 inhibitor GSK‐J4 (1 μM) treatment were assessed in terms of their miR‐155‐5p promoter activity with a luciferase reporter system. (J) PCa cells overexpressing EZH2 were treated with H3K27me3 inhibitor GSK‐J4 (1 μM), with RT‐qPCR employed for assessing the levels of miR‐155‐5p in PCa cells. *p < 0.05, **p < 0.01, ***p < 0.001, ****p < 0.0001. ns, no significant difference.

The PRC2 complex regulates promoter methylation, leading to the transcriptional repression of genes. EZH2 is a key member of the PRC2 complex, which can lead to the methylation modification of lysine 27 in histone. Our data showed that H3K27me3 methylation increased after EZH2 overexpression and decreased after EZH2 knockdown (Figure 2A,B). For demonstrating the regulatory effect of EZH2 on the miR‐155‐5p promoter, the activity of the promotor was detected utilizing a luciferase reporter system after EZH2 overexpression or knockdown in PCa cells. The finding demonstrated that an increase in EZH2 level considerably inhibits luciferase intensity, and EZH2 knockdown considerably increases luciferase intensity (Figure 2C). The effect of EZH2 on endogenous miR‐155‐5p expression was consistent with that of the luciferase reporter system (Figure 2D). In addition, a consistent polycomb response element was identified in the upstream region of miR‐155‐5p, indicating a possible binding region of the PRC2 complex (Figure 2E). Chromatin immunoprecipitation PCR results demonstrated that EZH2 can bind to the miR‐155‐5p promoter region (Figure 2F). GSK‐J4 is a potent H3K27me3/me2 demethylase, and H3K27me3 levels were substantially reduced by treatment with GSK‐J4 (1 μM) in PCa cells of the control group or EZH2 overexpressing group (Figure 2G,H). Similarly, treatment with GSK‐J4 (1 μM) further heightened the promotor activity of miR‐155‐5p (Figure 2I) and its expression levels (Figure 2J).

### 3.3 Tumor‐suppressive function of miR‐155‐5p in PCa

**FIGURE 3:**
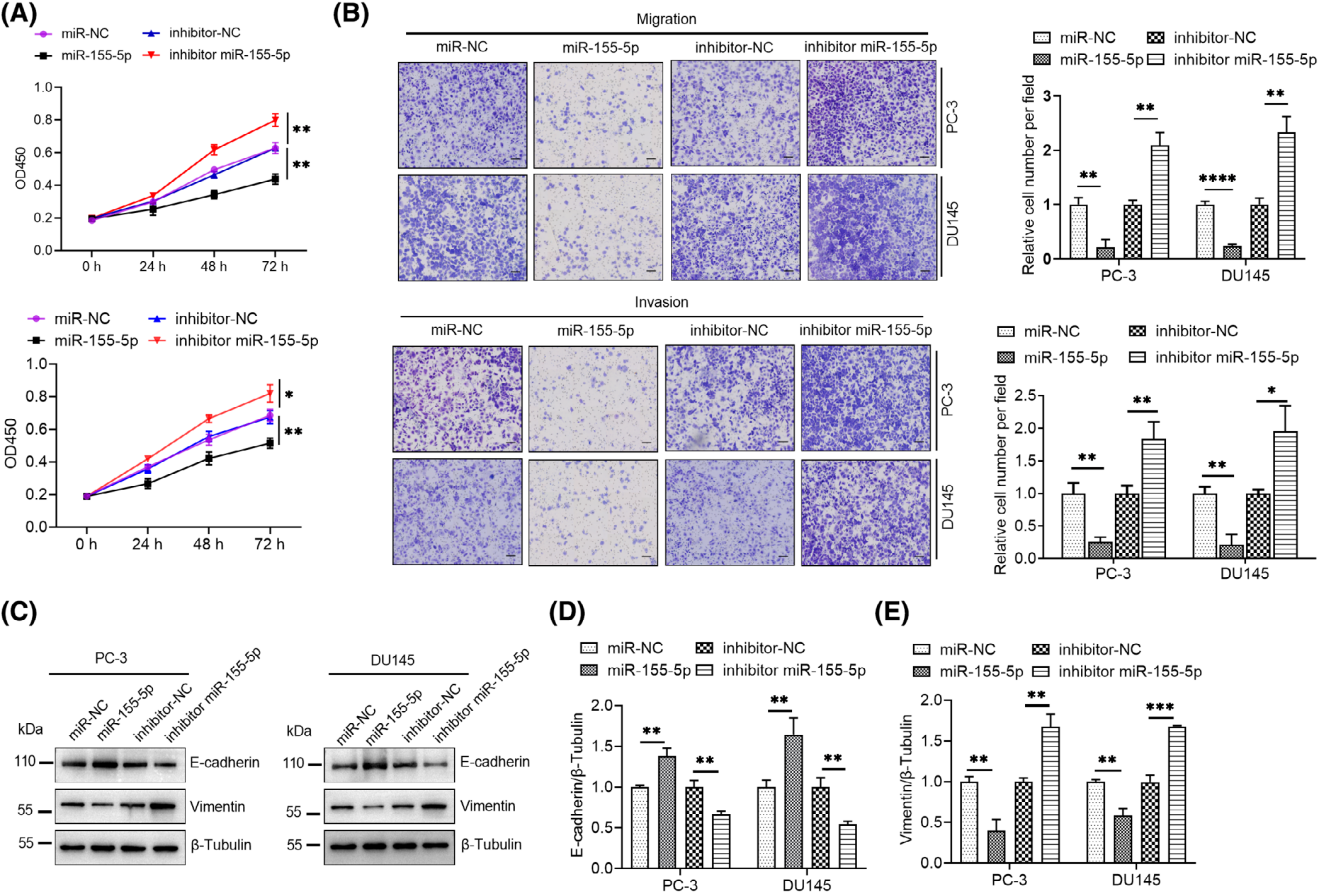
miR‐155‐5p inhibits the proliferation, migration and invasion of PCa cells. (A) PCa cells underwent transfection with miR‐155‐5p mimics (50 nM) or its inhibitor (50 nM), and the effect on cell viability was detected by CCK‐8. (B) Transwell assay was used to detect the effect of miR‐155‐5p expression level on the migratory and invasive capacities of PCa cells. (C) The expression levels of E‐cadherin and vimentin proteins were assessed by Western blot. *p < 0.05, **p < 0.01, ***p < 0.001, ****p < 0.0001.

The CCK‐8 outcomes revealed that the transfection of miR‐155‐5p mimics considerably lowered the proliferation of PCa cells. However, the transfection of miR‐155‐5p inhibitor caused the marked proliferation of PCa cells (Figure 3A). Moreover, miR‐155‐5p considerably diminished the migration and invasiveness of PCa cells, whereas its suppression enhanced their migration and invasion abilities (Figure 3B). Subsequent investigations revealed that miR‐155‐5p increased the expression of E‐cadherin and lowered that of vimentin and the suppression of miR‐155‐5p exhibited a contrasting trend (Figure 3C–E).

### 3.4 SMAD2 and TAB2 as downstream targets of miR‐155‐5p

**FIGURE 4:**
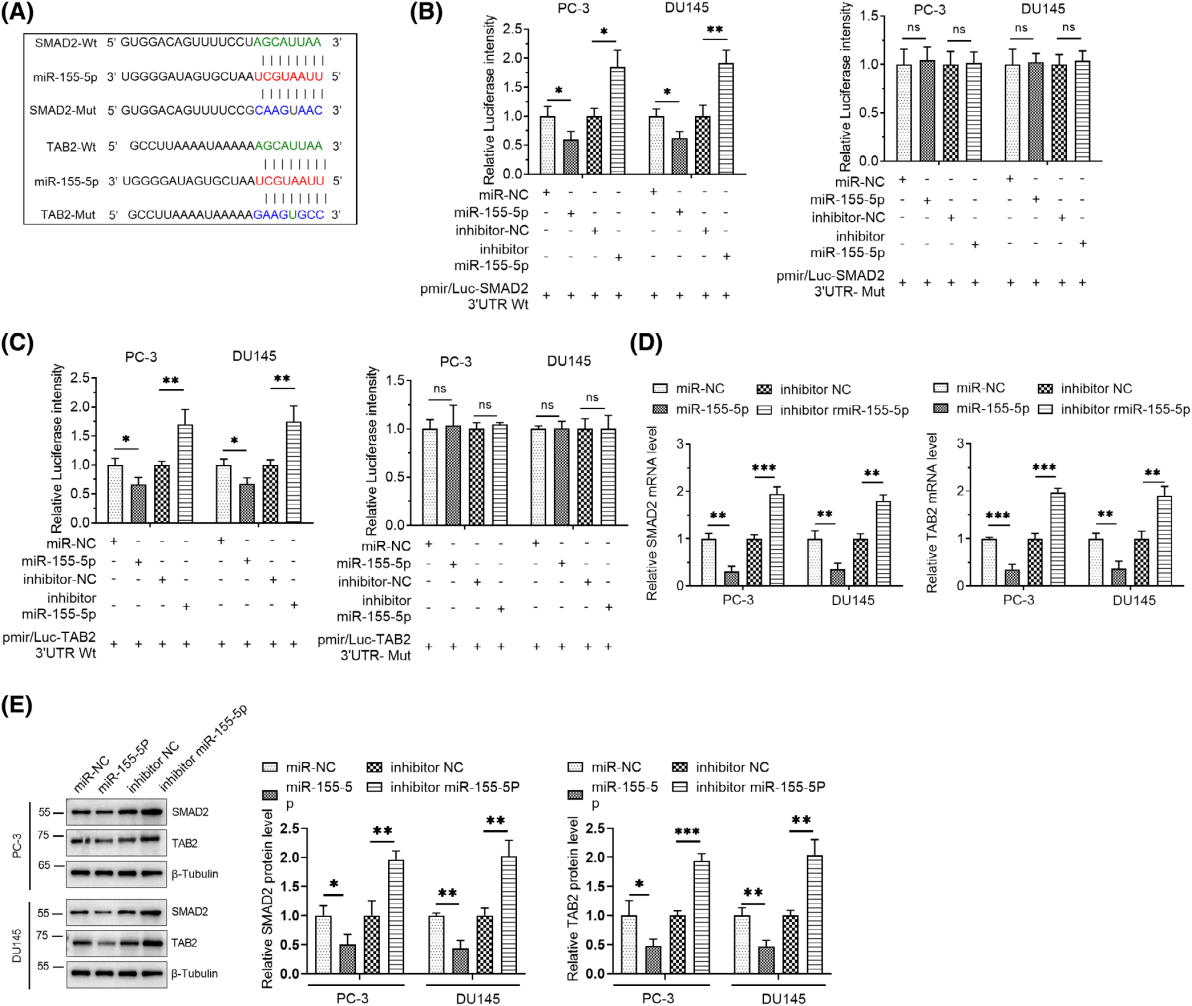
miR‐155‐5p targets the 3′UTRs of SMAD2 and TAB2. (A) TargetScan predicted the two targets of miR‐155‐5p, SMAD2 and TAB2. The wild‐type (Wt) and mutant (Mut) sequences of the two targets are shown in the figure. (B) Dual‐luciferase assay determined the relationship between miR‐155‐5p and SMAD2. (C) Dual‐luciferase assay assessed the relationship between miR‐155‐5p and TAB2. (D) RT‐qPCR detected the mRNA levels of SMAD2 and TAB2 after the expression level of miR‐155‐5p was changed. (E) Western blot detected the expression levels of SMAD2 and TAB2 after the expression level of miR‐155‐5p was changed. *p < 0.05, **p < 0.01, ***p < 0.001. ns, no significant difference.

miRNAs typically modulate associated biological effects by suppressing the expression of downstream targets. This investigation was conducted to predict potential targets for miR‐155‐5p, focusing on SMAD2 and TAB2 (Figure 4A). Mutations were introduced into the 3′UTRs of SMAD2 and TAB2, and the wild‐type 3′UTRs were cloned into fluorescent reporter vectors. Dual‐luciferase assay revealed that miR‐155‐5p directly interacted with the 3′UTRs of SMAD2 and TAB2, resulting in the inhibition of fluorescence intensity (Figure 4B,C). The RT‐qPCR results implied that miR‐155‐5p considerably lowered the transcription and translation of SMAD2 and TAB2. Conversely, the suppression of miR‐155‐5p led to a considerable elevation in the transcriptional and translational levels of SMAD2 and TAB2 (Figure 4D,E).

### 3.5 miR‐155‐5p regulation of the cellular phenotype of PCa through SMAD2 and TAB2

**FIGURE 5:**
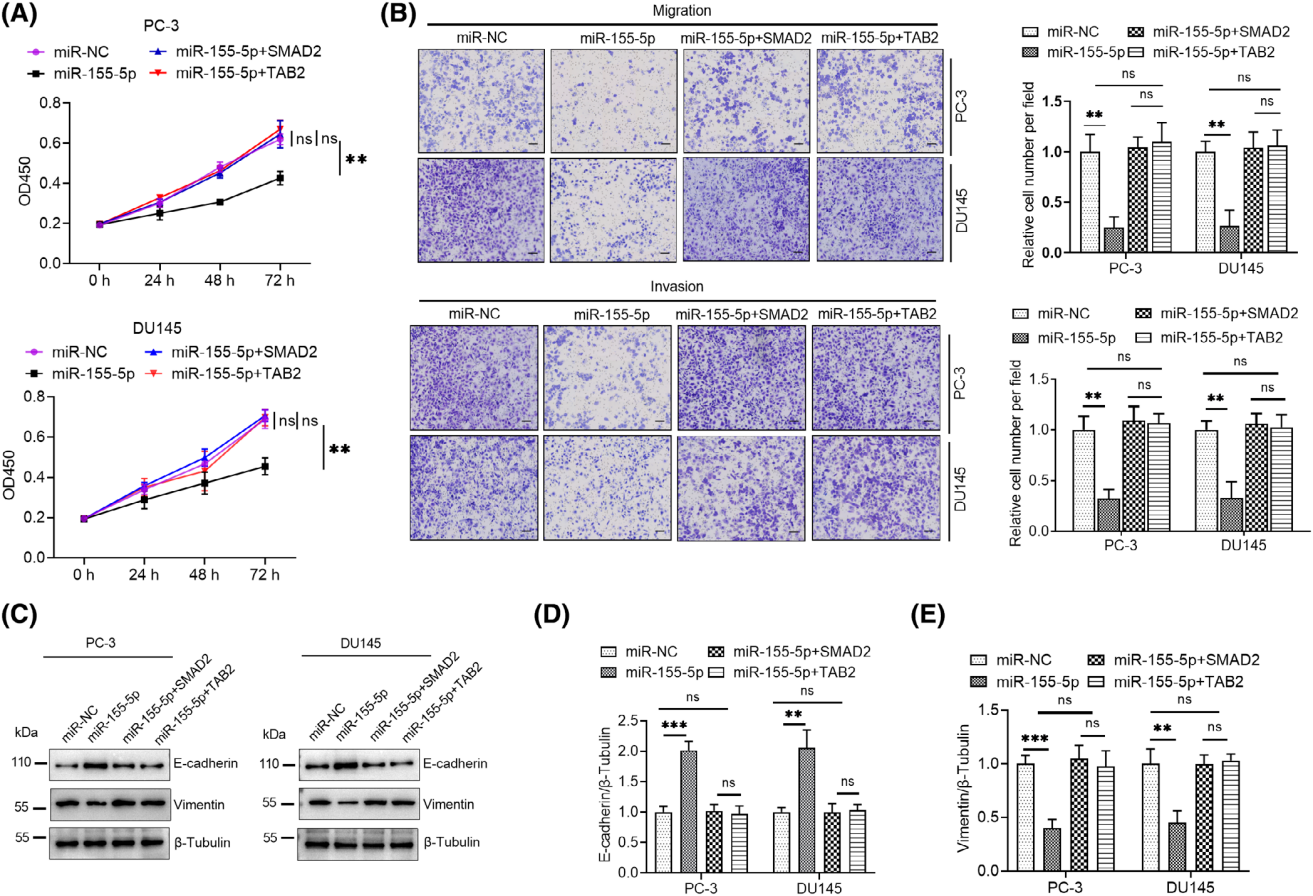
miR‐155‐5p suppresses the proliferation, migration and invasion of PCa cells by downregulating the expression of SMAD2 and TAB2. (A) PCa cells underwent transfection with miR‐155‐5p (50 nM), miR‐155‐5p (50 nM) + SMAD2 (1.5 μg) or miR‐155‐5p (50 nM) + TAB2 (1.5 μg), respectively, and effects on the proliferative capacity of the cells were examined via CCK‐8 assay. (B) Transwell assay was employed to investigate the influence of SMAD2 and TAB2 overexpression on miR‐155‐5p‐induced migration of and invasion by PCa cells. (C) The levels of vimentin and E‐cadherin assays were examined via Western blotting. *p < 0.05, **p < 0.01, ***p < 0.001, ****p < 0.0001. ns, no significant difference.

We demonstrated that miR‐155‐5p lowered the proliferation, invasion and migration capacity of PCa cells. Additionally, its two downstream targets, namely, SMAD2 and TAB2, were identified. The aim was to investigate whether the cellular phenotype of PCa is suppressed by miR‐155‐5p through downstream targets. To address this question, we simultaneously co‐transfected the overexpression plasmids for SMAD2 or TAB2 with miR‐155‐5p before CCK‐8 and Transwell assays. The outcomes were indicative of the capacity of miR‐155‐5p alone to diminish the proliferation, invasion and migration of PCa cells. This inhibitory effect was reversed when SMAD2 or TAB2 overexpression plasmids were co‐transfected (Figure 5A,B). Furthermore, Western blot assay suggested that the co‐transfection of miR‐155‐5p with an SMAD2 or TAB2 overexpression plasmid restored protein‐level changes induced by miR‐155‐5p (Figure 5C–E). These rescue experiments further elucidated that miR‐155‐5p regulates the phenotype of PCa cells through SMAD2 and TAB.

### 3.6 5‐Aza‐CdR regulated the phenotype of PCa cells via miR‐155‐5p

**FIGURE 6:**
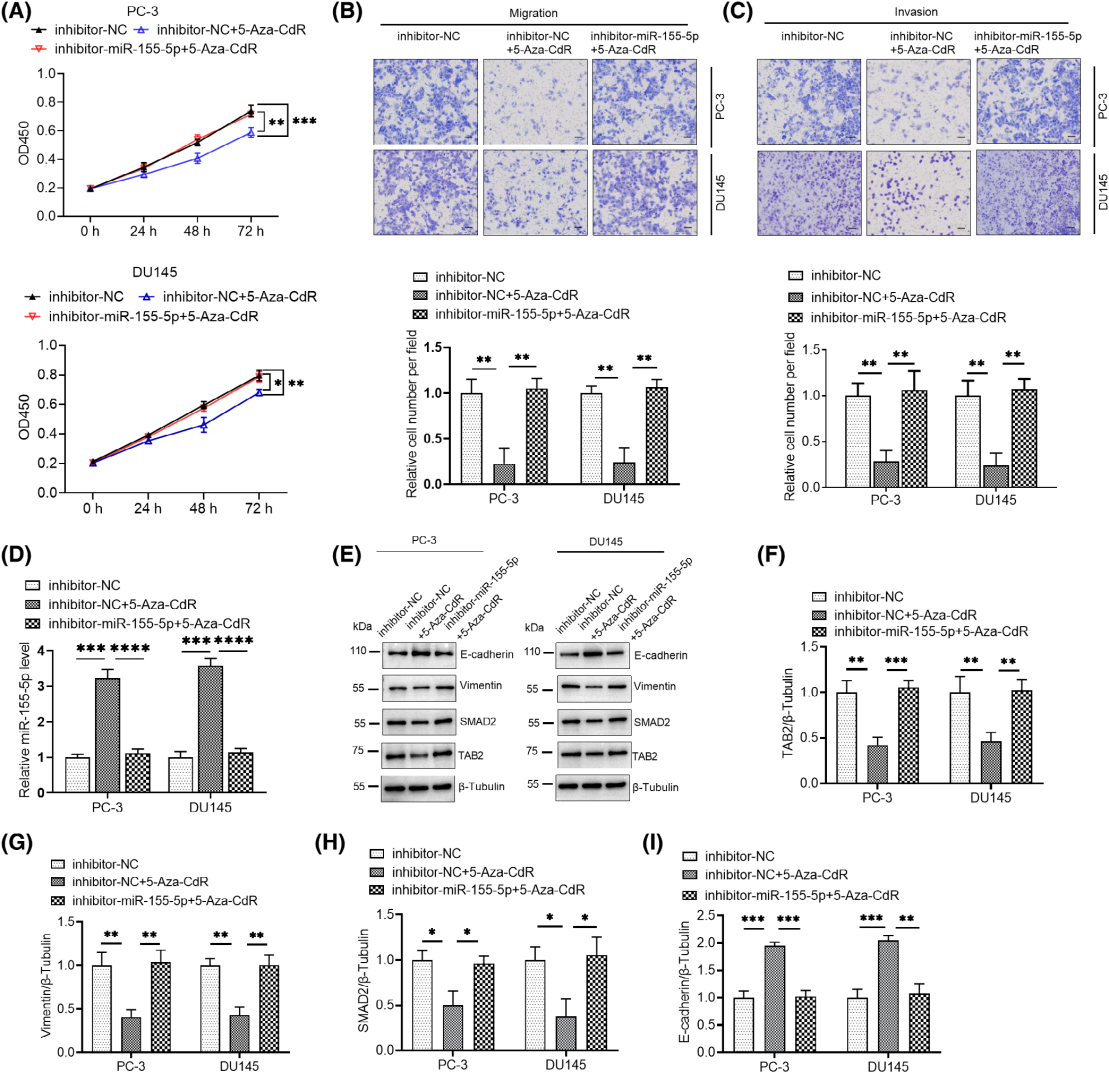
Downregulation of miR‐155‐5p expression alleviated the inhibitory effect of 5‐Aza‐CdR on PCa cells. (A) PCa cells were transfected with inhibitor‐NC (50 nM) or inhibitor‐miR‐155‐5p (50 nM) and then treated with 5‐Aza‐CdR (5 μM) according to the requirements in the figure. The effect on the proliferative capacity of PCa cells was assessed via CCK‐8 assay. (B and C) Transwell assay was used in assessing the effect of the downregulation of miR‐155‐5p expression level on PCa cell migration and invasion induced by 5‐Aza‐CdR (5 μM). (D) The levels of related proteins were examined through Western blot. (E–H) The levels of relevant proteins in (D) were quantified. *p < 0.05, **p < 0.01, ***p < 0.001. ns, no significant difference.

The function of 5‐Aza‐CdR in mediating the phenotype of PCa cells through miR‐155‐5p remains unclear. 5‐Aza‐CdR treatment reduced the proliferation, migration and invasion of PCa cells, and concurrent treatment with an inhibitor of miR‐155‐5p considerably increased these malignant behaviors (Figure 6A–C). Subsequent RT‐qPCR analysis exhibited a considerable elevation in the expression of miR‐155‐5p after 5‐Aza‐CdR treatment, whereas co‐treatment with a miR‐155‐5p inhibitor lowered the intracellular miR‐155‐5p (Figure 6D). Furthermore, SMAD2, TAB2 and vimentin expression levels were considerably reduced after 5‐Aza‐CdR treatment, and this effect was accompanied by an increase in E‐cadherin expression. Co‐treatment with a miR‐155‐5p inhibitor yielded similar changes but to a lesser extent (Figure 6E–I). These findings suggested that 5‐Aza‐CdR regulates the expression of miR‐155‐p by modulating its promoter methylation and subsequently influences the aggressive behavior of PCa cells.

## 4 DISCUSSION

Non‐coding RNA is closely associated with the onset, progression, metastasis and prognosis of PCa, exhibiting potential as a novel target for the early diagnosis and management of this disease.^17^, ^18^, ^19^ Specifically, miR‐155‐5p has emerged as a key player in the progression of cancer and has low expression levels in PCa, exerting a well‐documented effect that inhibits the malignant behavior of PCa cells.^9^, ^20^, ^21^ Given the differential expression patterns of miR‐155‐5p across various tumors, it is likely to exert context‐dependent roles either by promoting or suppressing tumorigenesis.^22^, ^23^, ^24^ However, the precise molecular mechanisms involved in the suppression of miR‐155‐5p in PCa are not fully elucidated.

This research confirmed through bioinformatics analysis that the suppression of miR‐155‐5p expression in PCa is linked to the methylation of its promoter. We synthesized the miR‐155‐5p promoter sequence and validated it by using a promoter‐luciferase reporter system and performing the 5‐Aza‐CdR treatment. The reduction in miR‐155‐5p expression in PCa was attributed to its promoter hypermethylation. As a crucial member of the PRC2 complex, EZH2 facilitates histone H3K27me3 and induces DNA methylation by recruiting DNA methylases.^25^, ^26^ Therefore, this investigation further examined whether EZH2 mediates the miR‐155‐5p promoter methylation. Additional experimental studies demonstrated that EZH2 can modulate the methylation level of the miR‐155‐5p promoter, and the overexpression of EZH2 enhances the methylation of the miR‐155‐5p promoter while downregulating inhibitory EZH2 promoter methylation. Furthermore, ChIP‐qPCR results indicated that EZH2 can bind to the miR‐155‐5p promoter. Treatment with GSK‐J4, a potent H3K27me3 inhibitor, increased miR‐155‐5p promotor activity and expression levels, providing further evidence of the involvement of EZH2‐mediated histone H3K27me3 in regulating miR‐155‐5p promotor methylation.

**FIGURE 7:**
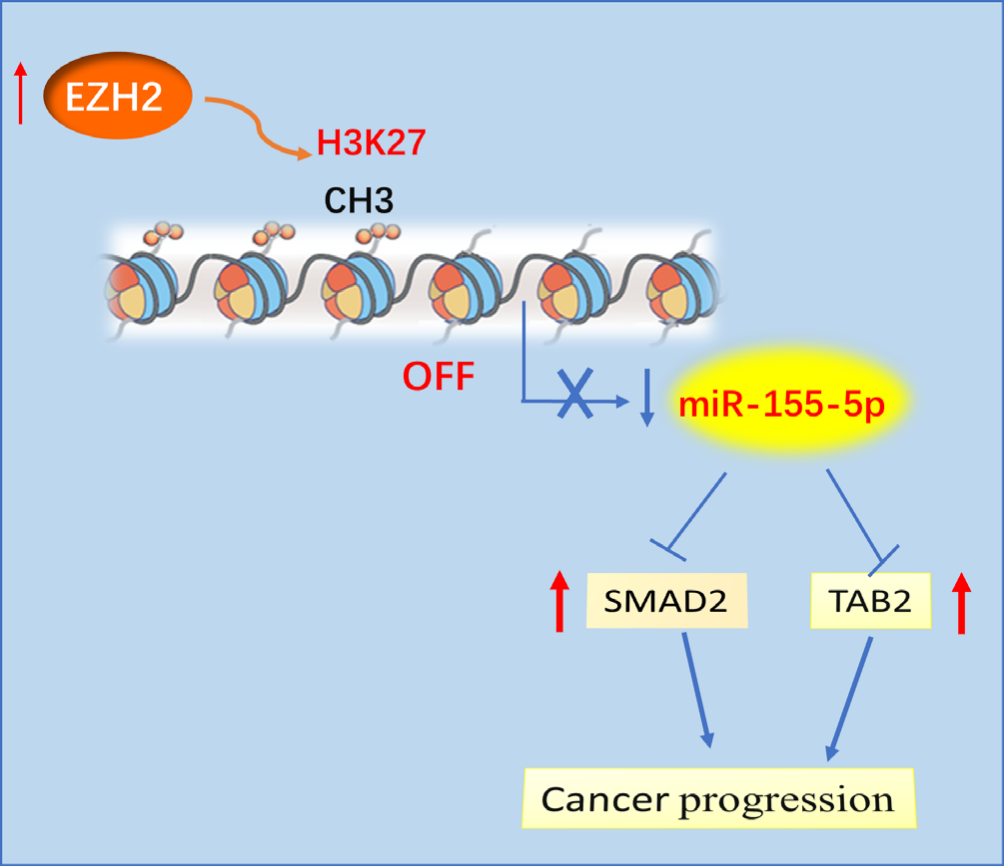
Schematic diagram of EZH2‐mediated downregulating of the miR‐155‐5p to promote PCa cell malignancy via targeting SMAD2 and TAB2.

In PCa, miR‐155‐5p inhibition tends to suppress cellular proliferation and EMT of PCa cells by reducing the expression of its marker protein vimentin and increasing E‐cadherin expression. These features allow for the suppression of cellular migration and invasion. Currently, several downstream targets of miR‐155‐5p have been documented, including SPOCK1,^20^ IKK‐ɛ,^21^ SMAD2,^21^ hMLH1^27^ and hMSH6.^27^ Apart from affirming the targeting relationship of miR‐155‐5p with SMAD2, we identified another novel downstream target, TAB2. miR‐155‐5p decreased the proliferative, migratory and invasive capacities of PCa cells by inhibiting SMAD2 and TAB2 expression. Finally, 5‐Aza‐CdR treatment for inhibiting miR‐155‐5p promoter methylation suppressed cellular proliferation, migration and invasion. This effect was reversed with a miR‐155‐5p inhibitor. In conclusion, this research essentially identified promoter methylation of miR‐155‐5p regulated by EZH2 as an important factor contributing to the low expression levels of miR‐155‐5 p in PCa cells, thus promoting the PCa cell malignancy through alleviating the suppression of SMAD2 and TAB2 (Figure 7).

## 5 CONCLUSION

EZH2 increased the proliferation, EMT, migration and invasion of PCa cells by promoting H3K27me3 methylation and repressing miR‐155‐5p. These effects in turn upregulated the downstream targets SMAD2 and TAB2. This study provides fresh perspectives on the onset and progression of PCa, offering valuable insights into the evolution and enhancement of molecularly targeted therapies for PCa.

## CONFLICT OF INTEREST STATEMENT

The authors declare that they have no conflict of interests.

## Supporting information


**APPENDIX S1:** Supplementary information.

## Data Availability

The datasets generated during and/or analyzed during the current study are available from the corresponding author upon reasonable request.

## References

[1] Siegel RL , Giaquinto AN , Jemal A . Cancer statistics, 2024. CA Cancer J Clin. 2024;74(1):12–49. 38230766 10.3322/caac.21820

[2] He S , Xia C , Li H , Cao M , Yang F , Yan X , et al. Cancer profiles in China and comparisons with the USA: a comprehensive analysis in the incidence, mortality, survival, staging, and attribution to risk factors. Sci China Life Sci. 2024;67(1):122–131. 37755589 10.1007/s11427-023-2423-1

[3] Schmid‐Alliana A , Schmid‐Antomarchi H , Al‐Sahlanee R , Lagadec P , Scimeca JC , Verron E . Understanding the progression of bone metastases to identify novel therapeutic targets. Int J Mol Sci. 2018;19(1):148. 29300334 10.3390/ijms19010148 PMC5796097

[4] Chung LW , Baseman A , Assikis V , Zhau HE . Molecular insights into prostate cancer progression: the missing link of tumor microenvironment. J Urol. 2005;173(1):10–20. 15592017 10.1097/01.ju.0000141582.15218.10

[5] Kang J , La Manna F , Bonollo F , Sampson N , Alberts IL , Mingels C , et al. Tumor microenvironment mechanisms and bone metastatic disease progression of prostate cancer. Cancer Lett. 2022;530:156–169. 35051532 10.1016/j.canlet.2022.01.015

[6] Zhang L , Wang W , Li X , He S , Yao J , Wang X , et al. MicroRNA‐155 promotes tumor growth of human hepatocellular carcinoma by targeting ARID2. Int J Oncol. 2016;48(6):2425–2434. 27035278 10.3892/ijo.2016.3465

[7] Liu X , Li Y , Li Z , Hou T . miR‐155 promotes proliferation and epithelial‐mesenchymal transition of MCF‐7 cells. Exp Ther Med. 2021;21(3):218. 33500705 10.3892/etm.2021.9650 PMC7818536

[8] Volinia S , Calin GA , Liu CG , Ambs S , Cimmino A , Petrocca F , et al. A microRNA expression signature of human solid tumors defines cancer gene targets. Proc Natl Acad Sci U S A. 2006;103(7):2257–2261. 16461460 10.1073/pnas.0510565103 PMC1413718

[9] Ji H , Li Y , Jiang F , Wang X , Zhang J , Shen J , et al. Inhibition of transforming growth factor beta/SMAD signal by MiR‐155 is involved in arsenic trioxide‐induced anti‐angiogenesis in prostate cancer. Cancer Sci. 2014;105(12):1541–1549. 25283513 10.1111/cas.12548 PMC4317958

[10] Dong GJ , Xu JL , Qi YR , Yuan ZQ , Zhao W . Critical roles of polycomb repressive complexes in transcription and cancer. Int J Mol Sci. 2022;23(17):9574. 36076977 10.3390/ijms23179574 PMC9455514

[11] Angrand PO . Structure and function of the polycomb repressive complexes PRC1 and PRC2. Int J Mol Sci. 2022;23(11):5971. 35682651 10.3390/ijms23115971 PMC9181254

[12] Blackledge NP , Klose RJ . The molecular principles of gene regulation by polycomb repressive complexes. Nat Rev Mol Cell Biol. 2021;22(12):815–833. 34400841 10.1038/s41580-021-00398-y PMC7612013

[13] Park SH , Fong KW , Mong E , Martin MC , Schiltz GE , Yu J . Going beyond polycomb: EZH2 functions in prostate cancer. Oncogene. 2021;40(39):5788–5798. 34349243 10.1038/s41388-021-01982-4 PMC8487936

[14] Wang R , Liu X . Epigenetic regulation of prostate cancer. Genes Dis. 2020;7(4):606–613. 33335960 10.1016/j.gendis.2019.10.018 PMC7729106

[15] Wee ZN , Li Z , Lee PL , Lee ST , Lim YP , Yu Q . EZH2‐mediated inactivation of IFN‐γ‐JAK‐STAT1 signaling is an effective therapeutic target in MYC‐driven prostate cancer. Cell Rep. 2014;8(1):204–216. 24953652 10.1016/j.celrep.2014.05.045

[16] Zeng Y , Xu Q , Xu N . Long non‐coding RNA LOC107985656 represses the proliferation of hepatocellular carcinoma cells through activation of the tumor‐suppressive Hippo pathway. Bioengineered. 2021;12(1):7964–7974. 34565286 10.1080/21655979.2021.1984005 PMC8806957

[17] Hua JT , Chen S , He HH . Landscape of noncoding RNA in prostate cancer. Trends Genet. 2019;35(11):840–851. 31623872 10.1016/j.tig.2019.08.004

[18] Al‐Rashidi RR , Noraldeen SAM , Kareem AK , Mahmoud AK , Kadhum WR , Ramírez‐Coronel AA , et al. Malignant function of nuclear factor‐kappaB axis in prostate cancer: molecular interactions and regulation by non‐coding RNAs. Pharmacol Res. 2023;194:106775. 37075872 10.1016/j.phrs.2023.106775

[19] Yang F , Li J , Ge Q , Zhang Y , Zhang M , Zhou J , et al. Non‐coding RNAs: emerging roles in the characterisation of immune microenvironment and immunotherapy of prostate cancer. Biochem Pharmacol. 2023;214:115669. 37364622 10.1016/j.bcp.2023.115669

[20] Yao LY , Ma J , Zeng XM , Ou‐Yang J . MicroRNA‐155‐5p inhibits the invasion and migration of prostate cancer cells by targeting SPOCK1. Oncol Lett. 2020;20(6):353. 33123264 10.3892/ol.2020.12215 PMC7586282

[21] Liao G , Ma H , Li Y , Sheng Y , Chen C . Selenium nanoparticles inhibit tumor metastasis in prostate cancer through upregulated miR‐155‐5p‐related pathway. Biosci Biotechnol Biochem. 2021;85(2):287–296. 33604641 10.1093/bbb/zbaa089

[22] Bayraktar R , Van Roosbroeck K . miR‐155 in cancer drug resistance and as target for miRNA‐based therapeutics. Cancer Metastasis Rev. 2018;37(1):33–44. 29282605 10.1007/s10555-017-9724-7

[23] Moutabian H , Radi UK , Saleman AY , Adil M , Zabibah RS , Chaitanya MNL , et al. MicroRNA‐155 and cancer metastasis: regulation of invasion, migration, and epithelial‐to‐mesenchymal transition. Pathol Res Pract. 2023;250:154789. 37741138 10.1016/j.prp.2023.154789

[24] Mattiske S , Suetani RJ , Neilsen PM , Callen DF . The oncogenic role of miR‐155 in breast cancer. Cancer Epidemiol Biomarkers Prev. 2012;21(8):1236–1243. 22736789 10.1158/1055-9965.EPI-12-0173

[25] Laugesen A , Højfeldt JW , Helin K . Molecular mechanisms directing PRC2 recruitment and H3K27 methylation. Mol Cell. 2019;74(1):8–18. 30951652 10.1016/j.molcel.2019.03.011 PMC6452890

[26] Liu X , Liu X . PRC2, chromatin regulation, and human disease: insights from molecular structure and function. Front Oncol. 2022;12:894585. 35800061 10.3389/fonc.2022.894585 PMC9255955

[27] Basu S , Majumder S , Bhowal A , Ghosh A , Naskar S , Nandy S , et al. A study of molecular signals deregulating mismatch repair genes in prostate cancer compared to benign prostatic hyperplasia. PLoS One. 2015;10(5):e0125560. 25938433 10.1371/journal.pone.0125560 PMC4418837

