# Supplementary material for: EZH2‐mediated downregulation of miR‐155‐5p contributes to prostate cancer cell malignancy through SMAD2 and TAB2
Source: Kaohsiung J Med Sci. 2025 Jan 9;41(3):e12936. doi: 10.1002/kjm2.12936 (PMC11924804; doi:10.1002/kjm2.12936)
Supplement: Supplementary file 1 — APPENDIX S1: Supplementary information. [file KJM2-41-e12936-s001.doc]

**Supplementary materials**

**Supplementary references of the 1500 bp upstream sequence of miR-155-5p transcript**

GGAACTGAGTTTGAATCTCAGCTTGACTACTTACTAGGATCTAAGGTAACATGTTTAATCTCTCTGTGCTCAGTTTCCTCATCTGGAAATAGAGATTATAATGCTCCTCCCTCATATGATCATAGTGAGGACTGAAAGAGTTAATGCATATAAGGAGTGTTAGGACAGGATCTGGCACATGGTAAATGCTTTATAAGTGTTAGTTGTTATCATCATGATTATTCTTGTTTGAATTATAAGAGAAAAAGCATGTATCTTAAGAATAGAGGGTTTTAAAATGCTCCCAAGTTCCTTAACCAACCTGAGCCATCTGTAAATTAAGTACTATGGGATTTCCAGCTCTGACATGTATTCTGACATGTAACTGGCATCAGTTTAATACAAACTATTCTAAAATGTCTTGTGCCCTTGACAGGGAGGTCCAAGTACTGGATACTTGCAATGCAATCCAGCAGTGGCCCCGTCTTTCTTACAAAAAGGCCCCAGTCACATGTTGATGAGGCTAGATCTATTCCTGTCCTCCTTCTCTTCCCCATATTTCCTTATTCTTCTTCAAGTCTAAACGTTTATTGAACTGAGACCCATGAATGAGTTACTCGACTAGGCTTGTAGGATAAACTTGCCAGGCTTTAGATCTATTTGTTCCTTTCCATCCCCCAAAATCAAATGGCTGCTCCAGGAAAATGTTCCCCTTGTGGCAGGGTCCGGGAGAAAAGAGAGAAGCGACAAAACCAAAAATTAAAACGACCGAAGTCCCATATGCTCCAGGAATATGTCCTGGAGATGGGAGTGGAGGGCAGGGGGAGAATGTTGTTGAGGTCAAAATTTTTGAAGTTTTAAGTCCTATATCTTGACATCCCGAGTATAAATGCGGGTACCAGACACAGTACAAACGTTCTCAAAGCCCAGTTACGTATTCCAAACCAAACGCGGGCTCTTGAAGGGTGATGAGGTAGGGATGAAATCCAGGATCGCCTGAAGACCATTTCTTCCTCTCTTAGGGACCTGCTGGTCTCCAGCTGATTCGGTCCAGGAGGAAAAACCTCCCACTTGCTCCTCTCGGGCTCCCTGCAAGGAGAGAGTAGAGACACTCCTGCCACCCAGTTGCAAGAAGTCGCCACTTCCCCCTCCAGCCGACTGAAAGTTCGGGCGACGTCTGGGCCGTCATTTGAAGGCGTTTCCTTTTCTTTAAGAACAAAGGTTGGAGCCCAAGCCTTGCGGCGCGGTGCAGGAAAGTACACGGCGTGTGTTGAGAGAAAAAAAATACACACACGCAATGACCCACGAGAAAGGGAAAGGGGAAAACACCAACTACCCGGGCGCTGGGCTTTTTCGACTTTTCCTTTAAAAAGAAAAAAGTTTTTCAAGCTGTAGGTTCCAAGAACAGGCAGGAGGGGGGAGAAGGGGGGGGGGGTTGCAGAAAAGGCGCCTGGTCGGTTATGAGTCACAAGTGAGTTATAAAAGGGTCGCACGTTCGCAGGCGCGGGCTTCCTGTGCGCG

Table S1 Sequences for miRNAs and inhibitors used in this study

| Name | Sequence (5’-3’) |
| --- | --- |
| miR-NC | UUCUCCGAACGUGUCACGUTT |
| miR-155-5p | UUAAUGCUAAUCGUGAUAGGG |
| inhibitor-NC | CAGUACUUUUGUGUAGUACAA |
| inhibitor miR-155-5p | CCCUAUCACGAUUAGCAUUAA |

Table S2 Sequences for RT-qPCR and CHIP-PCR primers used in this study

| Name | Sequence (5’-3’) |
| --- | --- |
| miR-155-5p RT primer | GTCGTATCCAGTGCAGGGTCCGAGGTGCACTGGATACGACACCCCTAT |
| U6-RT primer | GTCGTATCCAGTGCAGGGTCCGAGGTATTCGCACTGGATACGACAAAATATGGAAC |
| miR-155-5p-qPCR Forward | TGCGGGTTAATGCTAATCGTGA |
| miR-155-5p-qPCR Reverse | CCAGTGCAGGGTCCGAGGT |
| U6-qPCR Forward | TGCGGGTGCTCGCTTCGGCAGC |
| U6-qPCR Reverse | CCAGTGCAGGGTCCGAGGT |
| Smad2-qPCR Forward | CCGTTGCTCGACTCTAACCG |
| Smad2-qPCR Reverse | CCGACTTAGTGCCATTGGTAG |
| TAB2-qPCR Forward | GCAGCAAAGGAACATCTAGCC |
| TAB2-qPCR Reverse | TGGACTGTTAAGTACAGGTGGA |
| GAPDH -qPCR Forward | ACAACTTTGGTATCGTGGAAGG |
| GAPDH -qPCR Reverse | GCCATCACGCCACAGTTTC |
| ChIP-PCR Forward | AAGGGTGATGAGGTAGGGATGA |
| ChIP-PCR Reverse | CTACTCTCTCCTTGCAGGGAGC |
